# Increase of serotypes 15A and 23B in IPD in Germany in the PCV13 vaccination era

**DOI:** 10.1186/s12879-015-0941-9

**Published:** 2015-05-05

**Authors:** Mark van der Linden, Stephanie Perniciaro, Matthias Imöhl

**Affiliations:** Institute for Medical Microbiology, German National Reference Center for Streptococci, University Hospital RWTH Aachen, Pauwelsstr. 30, 52074 Aachen, Germany

**Keywords:** *Streptococcus pneumoniae*, Serotypes 15A and 23B, PCV13, Germany

## Abstract

**Background:**

This study presents an analysis of 1,491 serogroup 23 and 762 serogroup 15 isolates from invasive pneumococcal disease (IPD) in children and adults before and after the general recommendation for childhood pneumococcal conjugate vaccination in Germany in July 2006. Vaccination formulations used were PCV7 (from July 2006), PCV10 (from April 2009) and PCV13 (from December 2009, replacing PCV7).

**Methods:**

The German National Reference Center for Streptococci (GNRCS) has conducted surveillance of IPD since 1992. Isolates were serotyped and tested for antibiotic susceptibility. Selected isolates were characterized using MLST.

**Results:**

In an analysis of 23,957 isolates from IPD in children and adults sent to the GNRCS between July 1992 and June 2014, we found a strongly significant increase of non-PCV13 serotypes in the late vaccination (PCV13) period (2010-2014). Among these, the proportions of serotypes 15A and 23B were the most strongly significantly increasing. After the recommendation for pneumococcal conjugate childhood vaccination in 2006 and the introduction of higher-valent vaccines in 2009, the proportion of 15A increased significantly from 0.5% in the early vaccination period (2007-2010) to 2.4% in the late vaccination period (2010-2014, p=3.14x10^-22^). The proportion of serotype 23B increased from 0.5% to 2.8% in the same period (p=1.55x10^-29^). Penicillin non-susceptibility levels of the serotype 15A (47.4%) and serotype 23B (46.5%) isolates were high, with MIC values ranging from 0.12-2 μg/ml (15A) and 0.12-0.5 μg/ml (23B). MLSTs of serotype 23B isolates grouped in two clonal complexes (CC): CC439, with sequence type (ST) 439 as the main representative and CC338 (linked to CC156), with ST1349 as most prevalent clone. Both CCs have been present over almost the whole surveillance period. All penicillin non-susceptible isolates occurred in CC338. Serotype 15A isolates appeared to be more diverse. Six CCs, one group of three STs and two singletons were found among 20 isolates. Most prevalent was CC63, with ST63 as most prominent representative (n=5). Most penicillin non-susceptible isolates were found among CC63 isolates.

**Conclusions:**

The prevalence of non-PCV13 serotypes in Germany has increased significantly between July 2007 and June 2014, with 15A and 23B being the most strongly increasing serotypes of all. Both serotypes show a high proportion of penicillin non-susceptibility.

## Background

*Streptococcus pneumoniae* remains a major cause of infectious disease globally, especially in children. Invasive pneumococcal disease (IPD) causes over 1 million deaths among children worldwide [1]. The most important virulence factor of *S. pneumoniae* is the polysaccharide capsule on which pneumococcal vaccines are based.

A pneumococcal conjugate vaccine covering the seven serotypes most prevalent in IPD (4, 6B, 9V, 14, 18C, 19F and 23F; PCV7) has been available since 2001, and its use has resulted in a substantial reduction of vaccine type IPD in several countries where childhood vaccination programs were initiated [2-5].

However, an increase in serotypes not included in the vaccine (serotype replacement) was also observed. The most prominent replacement serotype was 19A, but increases in serotypes 1, 3 and 7F were also reported [6]. Introduction of higher-valent conjugate vaccines (PCV10: PCV7 serotypes + 1, 5, 7F) and PCV13: PCV10 serotypes + 3, 6A, 19A) has counteracted the increase of replacement serotypes, but once more bears the possibility of yet another round of serotype replacement. Indeed, reports from several countries have described increases of different non-PCV13 serotypes in both carriage and IPD [7,8], but so far no particular serotypes have become apparent.

In Germany, general childhood pneumococcal conjugate vaccination started in July 2006 when a vaccination recommendation for all children under the age of two years was issued by the German Standing Committee on Vaccination (STIKO). Vaccination began with a seven-valent vaccine (Prevenar®; PCV7), followed in 2009 by both a ten-valent vaccine (Synflorix®, PCV10) and a 13-valent vaccine (Prevenar13®, PCV13), with PCV13 replacing PCV7. Vaccination is fully reimbursed by health insurance and the choice of vaccine resides with the parents/paediatrician. Currently, more than 95% of all children in Germany that are vaccinated with a pneumococcal conjugate vaccine are vaccinated with PCV13.

Childhood pneumococcal vaccination has led to strong effects on the serotype distribution among IPD in vaccinated children. The incidence of PCV7-serotype IPD has decreased strongly. Although an increase in non-PCV7 serotypes was apparent, a net reduction in IPD was observed [3,9]. After the introduction of higher-valent vaccination, a further reduction of IPD caused by PCV13 serotypes was observed. Also among non-vaccinated children and among adults, the prevalence of vaccine serotypes strongly decreased (herd protection).

Analysis of non-PCV13 serotypes in the early vaccination (PCV7) period and late vaccination (PCV13) period showed that two serotypes, 15A and 23B were by far the most higly significantly increasing serotypes in the late vaccination period. The current study describes this highly-significant increase of serotypes 15A and 23B among IPD in children and adults in the late vaccination (i.e. PCV13) period in Germany.

## Methods

### Study material

The German National Reference Center for Streptococci (GNRCS) has conducted surveillance for IPD in Germany since 1992, using a laboratory-based approach to collect data about IPD among children (<16 years) and adults (≥16 years). Microbiological diagnostic laboratories from all over Germany were requested to send isolates of invasive pneumococcal disease to the GNRCS. Currently, over 300 laboratories participate, including the large, nationally-operating commercial labs. Over the years, the surveillance system has been improved. In 2001, surveillance for adults was enhanced in North Rhine-Westphalia (22% of German population), as well as in Bavaria and Saxony in 2006. On each occasion, all laboratories in the respective federal states were approached and asked to send in isolates. In 2007, a web-based surveillance system (Pneumoweb) was set-up in collaboration with the Robert Koch Institute. Pneumoweb enables the laboratories to report a case of IPD via an online reporting system, and at the same time, print the reported information to accompany the IPD isolate, which can then be sent to the GNRCS. The web-based system resulted in a large increase in reported cases for adults, whereas the amount of cases for children remained at the same high level.

Invasive pneumococcal disease cases were defined as *S. pneumoniae* isolates from blood, cerebrospinal fluid (CSF) or any other normally sterile body fluid. Cases were grouped per pneumococcal season (from July to June of the consecutive year) because of known infection clusters during winter.

### Pneumococcal vaccination

A recommendation for pneumococcal conjugate vaccination for all children under 2 years of age was issued by the STIKO in July 2006. Germany does not have a national immunization plan, but vaccination costs of recommended vaccinations are reimbursed by health insurance companies. A seven-valent pneumococcal conjugate vaccine (PCV7) was licensed in Germany in 2001, followed by) in April 2009 and PCV13 in December 2009 (replacing PCV7). The choice of vaccine is made by the parents and the pediatrician.

### Serotyping

Pneumococcal isolates were serotyped by Neufeld’s Quellung reaction using type and factor sera provided by the Statens Serum Institut, Copenhagen, Denmark.

### Susceptibility testing

All strains were tested for antibiotic minimal inhibitory concentrations (MIC) using the broth microdilution method as recommended by the CLSI [10]. The microtiter plates (Sensititre NLMMCS10, TREK Diagnostic Systems Ltd., East Grinstead, UK) contained penicillin G (PEN), cefotaxime (CEF), clarithromycin/erythromycin (CLA/ERY), clindamycin (CLI), tetracycline (TET), levofloxacin (LEV), chloramphenicol (CHL) and trimethoprim/sulfamethoxazole (SXT) with cation-adjusted Mueller-Hinton broth (Oxoid, Wesel, Germany) and 5% lysed horse blood. Macrolide resistance testing was performed using erythromycin from 1992-2003 and from 2011-2014. From 2004 to 2010, clarithromycin was used. The current CLSI criteria were applied for interpretation [10]. To assess the development of penicillin resistance and in the definition of multidrug resistance, the ‘oral’ penicillin breakpoints were used (≤0.06μg/ml, 0.12-1μg/ml, ≥2μg/ml), since they give better insight into resistance development over time. Breakpoints used for other antibiotics were: clarithromycin/erythromycin, clindamycin: ≤0.25μg/ml, 0.5μg/ml, ≥1μg/ml, tetracycline: ≤1μg/ml, 2μg/ml, ≥4μg/ml, levofloxacin: ≤2μg/ml, 4μg/ml, ≥8μg/ml, chloramphenicol ≤4μg/ml, ≥8μg/ml and trimethoprim/sulfamethoxazole ≤0.5/9.5μg/ml, 1/19-2/38μg/ml, ≥4/76μg/ml. Isolates were considered multidrug-resistant when they were resistant to more than two different classes of antibiotics.

### Multilocus sequence typing

Multilocus sequence typing of selected pneumococcal isolates was performed as described previously [11]. Briefly, internal fragments of the *aroE, gdh, gki, recP, spi, xpt,* and *ddl* genes were amplified by PCR from chromosomal DNA with the described primer pairs. A special allelic profile is provided by the alleles at each of the seven loci and their sequence type (ST) is defined. The allelic profiles were compared with each other and with other isolates in the pneumococcal MLST database using software available at http://pubmlst.org/spneumoniae/. Clusters of related STs were grouped into clonal complexes (CCs) using the program PHYLOViZ on the global database on http://pubmlst.org/spneumoniae/ [12].

### Statistical methods

Differences in proportions were tested by Fisher’s exact test with a two-sided P value of <0.05 considered significant. Analyses were conducted using R (R Foundation for Statistical Computing, Vienna, Austria, 2014).

### Ethical statement

An ethical approval was not required since the study was performed with *Streptococcus pneumoniae* isolates that resulted from routine microbiological diagnostic procedures as requested by the treating physician. No additional biological specimens were taken for the purpose of this study. Specimens were anonymized and only data on year and month of birth, sex, type of specimen and hospital/laboratory where the case was diagnosed were registered.

## Results

From July 1992 until June 2014, a total of 3,853 isolates from IPD among children and 20,104 isolates from IPD among adults were sent to the German National Reference Center for Streptococci. Among these isolates 1,491 (6.2%) belonged to serogroup 23 and 762 (3.2%) to serogroup 15.

**Figure 1:**
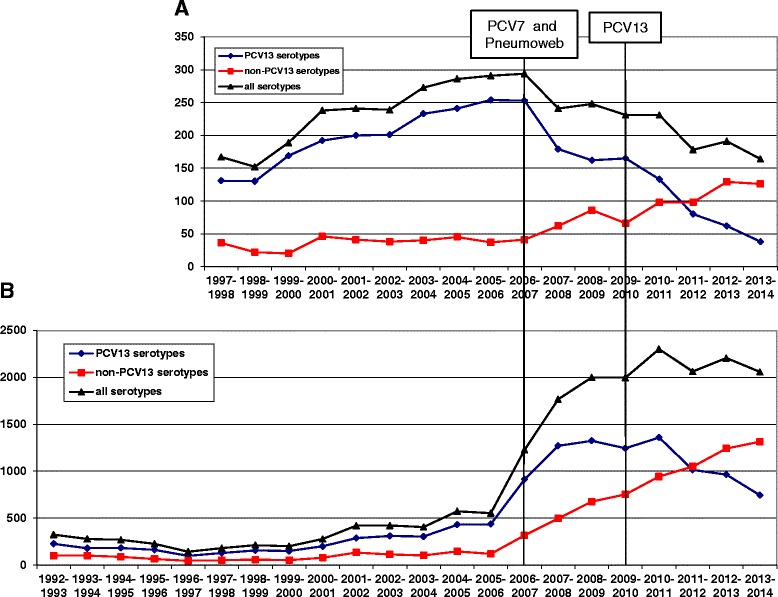
Numbers of IPD cases reported to the GNRCS per pneumococcal season (July in one year - June in the following year), for children **(A)** and adults **(B)**.

Since the start of childhood pneumococcal conjugate vaccination, the number of reported cases of IPD among children has decreased by about 50% (Figure 1A). The prevalence of the 13 vaccine serotypes strongly decreased after the start of PCV7 vaccination (due to the reduction of the seven serotypes included in this vaccine) but this decrease leveled off in 2009-2010. With the introduction of higher-valent vaccines, a further strong decrease was observed, which continues to date. Non-PCV13 serotypes increased both after the start of PCV7 vaccination as well as after the introduction of higher-valent vaccination (Figure 1A).

Among adults, a strong increase of reported cases was observed in 2006-2007, when a new reporting system (Pneumoweb) was introduced. From 2007-2010, PCV13 serotypes remained at the same level, with PCV7 serotypes decreasing, but PCV13-non-PCV7 serotypes increasing. Starting from 2011-2012, a decrease in PCV13 cases was observed, which was off-set by a similar increase in non-PCV13 serotypes (Figure 1B).

The most strongly increasing non-PCV13 serotypes in the late vaccination period included 12F, 23B, 24F and 38 among children and 6C, 12F, 15A, 23B and 22F among adults. Among these 15A and 23B were by far the most strongly significantly increasing serotypes.

As a result of childhood pneumococcal conjugate vaccination (PCV7 and PCV10/13), the prevalence of vaccine serotypes has decreased considerably both among children as well as among adults. PCV7 serotype prevalence among children was reduced from 61.8% in the pre-vaccination period (1992-2006) to 23.5% in the PCV7 vaccination period (2007-2010; p < 0.05) and 5.2% in the PCV10/13 vaccination period (2010-2014; p < 0.05; Table 1). Among adults, PCV7 serotype prevalence was 43.4% prior to childhood vaccination, and fell to 24.7% (p < 0.05) and 8.2% (p < 0.05), in the respective vaccination periods. Similarly, the prevalence of PCV13 serotypes decreased from 84.4% to 70.3% (p < 0.05) and 40.8% (p < 0.05) among children and from 72.3% to 66.6% (p < 0.05) and 47.3% (p < 0.05) among adults (Table 1).

**Table 1:** **Effects of PCV7 and PCV13 vaccination on IPD among children (0-15 years) and adults (≥16 years) in Germany**

**Serotype**	**Age group**	**Pre-vaccination (1992-2006)**		**Early vaccination (PCV7) (2007-2010)**		**Late vaccination (PCV13) (2010-2014)**		**Pre-vaccination vs. Early vaccination**	**Early vaccination vs. Late vaccination**
		**n=**	**%**	**n=**	**%**	**n=**	**%**	**p value**	**p value**
PCV7	children	1,282	61.8	169	23.5	40	5.2	**1,30E-72**	**4,59E-25**
non-PCV7	children	793	38.2	551	76.5	724	94.8		
all	children	2,075	100.0	720	100.0	764	100.0		
PCV7	adults	1,943	43.4	1,426	24.7	711	8.2	**3.78E-88**	**5.97E-161**
non-PCV7	adults	2,534	56.6	4,338	75.3	7,925	91.8		
all	adults	4,477	100.0	5,764	100.0	8,636	100.0		
PCV7	all	3,225	49.2	1,595	24.6	751	8.0	**4.39E-189**	**2.06E-182**
non-PCV7	all	3,327	50.8	4,889	75.4	8,649	92.0		
all	all	6,552	100.0	6,484	100.0	9,400	100.0		
PCV13	children	1,751	84.4	506	70.3	312	40.8	**1.50E-15**	**1.96E-30**
non-PCV13	children	324	15.6	214	29.7	452	59.2		
all	children	2,075	100.0	720	100.0	764	100.0		
PCV13	adults	3,238	72.3	3,839	66.6	4,086	47.3	**5.24E-10**	**1.90E-116**
non-PCV13	adults	1,239	27.7	1,925	33.4	4,550	52.7		
all	adults	4,477	100.0	5,764	100.0	8,636	100.0		
PCV13	all	4,989	76.1	4,345	67.0	4,398	46.8	**5.55E-31**	**9.74E-142**
non-PCV13	all	1,563	23.9	2,139	33.0	5,002	53.2		
all	all	6,552	100.0	6,484	100.0	9,400	100.0		
23A	all	37	0.6	101	1.6	225	2.4	**2.45E-08**	**2.57E-04**
23B	all	10	0.2	34	0.5	265	2.8	**2.26E-04**	**1.55E-29**
23F	all	407	6.2	205	3.2	93	1.0	**1.36E-16**	**1.29E-22**
15A	all	45	0.7	35	0.5	226	2.4	3.13E-01	**3.14E-22**
15B	all	38	0.6	79	1.2	120	1.3	**1.22E-04**	7.72E-01
15C	all	33	0.5	41	0.6	105	1.1	3.52E-01	**1.66E-03**
15B/C	all	71	1.1	120	1.9	225	2.4	1.25E-01	6.55E-01
15F	all	6	0.1	1	0.0	4	0.0	**3.31E-04**	**2.31E-02**

The pneumococcal season 2006-2007 in which childhood pneumococcal conjugate vaccination was introduced was considered a transition period and left out of the analysis. P-values reaching statistical significance are depicted in bold.

In serogroup 23, serotype 23F, which is contained in all pneumococcal conjugate vaccine formulations, decreased from 6.2% to 1.0% (p < 0.05). The two other members of this serogroup, however, increased in prevalence (23A: from 0.6% to 2.4% (p < 0.05); 23B from 0.2% to 2.8%; (p < 0.05; Table 1).

**Figure 2:**
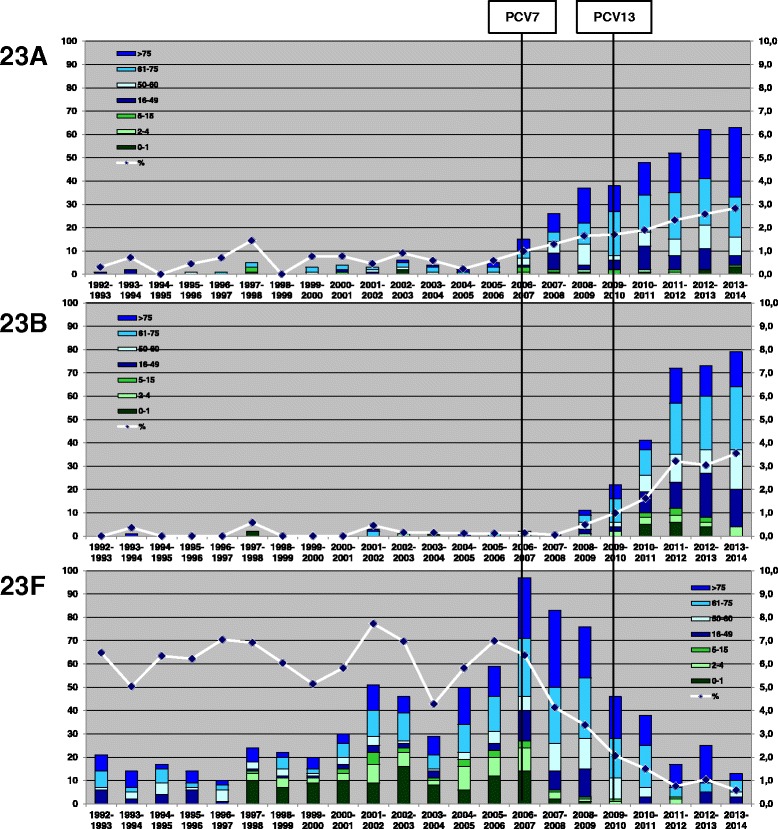
Numbers of reported IPD cases with serogroup 23 serotypes for children (0-1, 2-4 and 5-15 years of age) and adults (16-49, 50-60, 61-75 and >75 years of age (bars, left vertical axis), and percentage of all isolates (line, right vertical axis).

Serotype 23F was among the most prevalent serotypes among IPD in children in the pre-vaccination period, with up to 30 cases per season, mostly among children under the age of 2 years. Among adults, 23F was highly prevalent in all four age groups (16-49, 50-60, 61-75 and >75 years). In 2013-2014, 23F disappeared among children, and among adults only 13 cases were reported (Figure 2).

Serotype 23A was rarely reported in the pre-vaccination period. After the start of vaccination, its prevalence increased gradually, starting from 2006-2007 and mostly among adults (Figure 2).

Serotype 23B was even less prevalent than serotype 23A before the start of vaccination. In 2008-2009, two seasons after the introduction of PCV7, reports of serotype 23B strongly increased, and this increase continued till 2011-2012. The increase in reported cases was observed in all age groups. In 2013-2014, a less strong increase in serotype 23B cases was observed among adults, whereas a decrease in reported cases was seen among children (Figure 2).

In serogroup 15, an increase in prevalence was seen for serotype 15A but only in the late vaccination period (2010-2014; 0.5% to 2.4%, p < 0.05), and, to a lesser extent, for serotypes 15B/C (1.1% to 2.4%). Serotype 15F was very rare in Germany, with only 12 reported cases in 22 seasons of surveillance (1992-2014, with one case reported in the transition season 2006-2007; Table 1).

**Figure 3:**
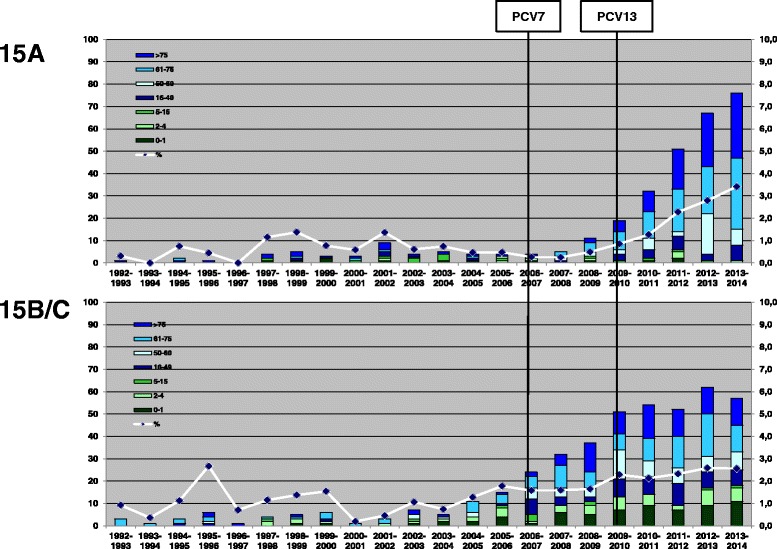
Numbers of reported IPD cases with serogroup 15 serotypes for children (0-1, 2-4 and 5-15 years of age) and adults (16-49, 50-60, 61-75 and >75 years of age (bars, left vertical axis), and percentage of all isolates (line, right vertical axis).

Reports of cases with serotypes 15B/C were rare in the pre-vaccination period. After the start of vaccination, more cases were reported resulting in a prevalence increase from 1.1% to 2.4% (p < 0.05). The increase was observed in all age groups (Table 1, Figure 3).

Serotype 15A cases were also rarely reported before vaccine introduction. Starting from 2008-2009, a strong increase in reported cases was observed, which continues to date, and seems to be most prominent among adults. The prevalence of serotype 15A has strongly increased in the late vaccination period, from about 0.5% (2008-2009) to 3.4% currently (2013-2014; p < 0.05). Serotype 15A was most often present among adults (Figure 3).

Antibiotic resistance levels of the serotype 15A and serotype 23B isolates are listed in Table 2. Among serotype 15A isolates, 47.4% were penicillin non-susceptible (PenNS), but all were susceptible to cefotaxime. The 15A isolates presented high rates of resistance to macrolides (48.7%), clindamycin (46.5%) and tetracycline (44.8%). Towards trimethoprim/sulfamethoxazole, 1.8% of the isolates were resistant whereas 5.6% showed intermediate resistance. Only 1.1% of isolates were chloramphenicol resistant and no resistance to levofloxacin was found. Multi-drug resistance was found in 145 out of 310 isolates (46.8%). Most MDR isolates (n = 128) were PenNS, and ERY, CLI and TET resistant. Others were PenNS and ERY and CLI resistant (n = 8), ERY, CLI, TET and SXT resistant (n = 3), PenNS and ERY, CLI, TET and CHL resistant (n = 2) and ERY, CLI and TET resistant (n = 1).

**Table 2:** **Antibiotic resistance profiles for isolates with serotype 23B and serotype 15A**

	**Serotype 15A**						**Serotype 23B**					
	**all**	**MIC range**	**I**	**R**	**MIC 50**	**MIC 90**	**all**	**MIC range**	**I**	**R**	**MIC 50**	**MIC 90**
	**n=**	**μg/ml**	**%**	**%**	**μg/ml**	**μg/ml**	**n=**	**μg/ml**	**%**	**%**	**μg/ml**	**μg/ml**
**Penicillin**	310	0.015 - 2	47.1	0.3	0.015	0.25	312	0.015 - 0.5	46.5	0.0	0.015	0.25
1992-1993	1	0.015	0.0	0.0	0.015	0.015	0					
1993-1994	0						1	0.015	0.0	0.0	0.015	0.015
1994-1995	2	0.015	0.0	0.0	0.015	0.015	0					
1995-1996	1	0.015	0.0	0.0	0.015	0.015	0					
1996-1997	0						0					
1997-1998	4	0.015 - 0.12	50.0	0.0	0.015	0.12	2	0.015 - 0.06	0.0	0.0	0.015	0.06
1998-1999	5	0.015 - 0.25	20.0	0.0	0.015	0.25	0					
1999-2000	3	0.015 - 0.25	33.3	0.0	0.015	0.25	0					
2000-2001	3	0.015 - 0.12	33.3	0.0	0.03	0.12	0					
2001-2002	9	0.015	0.0	0.0	0.015	0.015	3	0.015 - 0.12	33.3	0.0	0.015	0.12
2002-2003	4	0.015 - 0.25	25.0	0.0	0.015	0.25	1	0.015	0.0	0.0	0.015	0.015
2003-2004	5	0.015	0.0	0.0	0.015	0.015	1	0.25	100.0	0.0	0.25	0.25
2004-2005	4	0.015 - 0.25	25.0	0.0	0.015	0.25	1	0.015	0.0	0.0	0.015	0.015
2005-2006	4	0.015 - 0.06	0.0	0.0	0.015	0.06	1	0.015	0.0	0.0	0.015	0.015
2006-2007	4	0.015	0.0	0.0	0.015	0.015	2	0.015	0.0	0.0	0.015	0.015
2007-2008	5	0.015 - 0.5	40.0	0.0	0.015	0.5	1	0.015	0.0	0.0	0.015	0.015
2008-2009	11	0.015 - 0.5	45.5	0.0	0.015	0.5	11	0.015 - 0.5	45.5	0.0	0.015	0.25
2009-2010	19	0.015 - 1	47.4	0.0	0.03	0.5	22	0.015 - 0.5	59.1	0.0	0.12	0.25
2010-2011	32	0.015 - 0.5	56.3	0.0	0.25	0.25	41	0.015 - 0.5	39.0	0.0	0.015	0.25
2011-2012	51	0.015 - 0.5	52.9	0.0	0.12	0.25	72	0.015 - 0.25	44.4	0.0	0.06	0.25
2012-2013	67	0.015 - 1	61.2	0.0	0.25	0.25	73	0.015 - 0.5	52.1	0.0	0.12	0.25
2013-2014	76	0.015 - 2	50.0	1.3	0.25	0.25	80	0.015 - 0.5	48.8	0.0	0.015	0.25
**Cefotaxime**	310	0.015 - 1	0.0	0.0	0.015	0.12	312	0.015 - 0.5	0.0	0.0	0.03	0.06
**Erythromycin**	310	0.06 - 256	0.3	48.7	0.12	256	312	0.06 - 256	0.0	2.9	0.12	0.12
**Clindamycin**	310	0.06 - 256	0.6	46.5	0.12	128	312	0.06 - 256	0.0	1.0	0.12	0.12
**Tetracycline**	310	0.12 - 128	0.0	44.8	0.5	64	311	0.03 - 128	0.0	1.6	0.5	0.5
**Levofloxacin**	284	0.5 - 2	0.0	0.0	1	2	305	0.25 - 8	0.3	0.3	1	1
**Chloramphenicol**	285	0.5 - 4	0.0	1.1	4	4	307	0.5 - 4	0.0	0.0	4	4
**Trimethoprim/Sulfamethoxazole**	284	0.12 - 8	5.6	1.8	0.25	0.5	305	0.25 - 8	24.3	18.0	0.25	4
**MDR**	310			46.8			312			1.3		

Serotype 23B isolates were PenNS in 46.5% of the cases, and all of these isolates were also cefotaxime susceptible. The non-susceptibility rate to trimethoprim/sulfamethoxazole was 42.3% (18.0% resistant and 24.3% intermediate resistant). Rates of resistance to macrolides (2.9%), clindamycin (1.0%), tetracycline (1.6%) and levofloxacin (0.3%) were very low. All isolates were susceptible to chloramphenicol. Multi-drug resistance was found in only 4 out of 312 isolates (1.3%). Two isolates were PenNS and ERY, CLI and TET resistant, one ERY, CLI, TET and SXT resistant and one ERY, TET and SXT resistant.

Serotype 23B PenNS isolates have MICs up to 0.5 μg/ml, but most PenNS cases have an MIC of 0.25 μg/ml. Among the 15A PenNS isolates, most have an MIC of 0.25 μg/ml as well. However, several cases with MICs of 1 and 2 μg/ml have appeared in the last two seasons (Table 2).

When looking at the serotype ranking of PenNS isolates, before the start of vaccination the first six places were occupied by serotypes 14, 6B, 9V, 19A, 23F and 19F (all PCV7 serotypes, except for 19A (PCV13)). In the PCV7-vaccination years, PCV7 serotypes gradually disappeared from the top ten, and serotype 19A became the main serotype associated with penicillin non-susceptibility. Also, serotypes 15A and 23B appeared among the top five PenNS serotypes. In the PCV13-vaccination period, serotypes 15A and 23B are the most prevalent PenNS serotypes, and 19A appeared only in third place (Table 3).

**Table 3:** **Ranking of penicillin non-susceptible (MIC >0.06 μg/ml) isolates from IPD among children and adults in Germany before childhood vaccination (1992-2006) and after PCV7 (2006) and PCV10/PCV13 (2009) vaccination**

**PenNS rank**	**1992-2006**	**n=**	**%**	**2006-2007**	**n=**	**%**	**2007-2008**	**n=**	**%**	**2008-2009**	**n=**	**%**	**2009-2010**	**n=**	**%**	**2010-2011**	**n=**	**%**	**2011-2012**	**n=**	**%**	**2012-2013**	**n=**	**%**	**2013-2014**	**n=**	**%**
1	14	66	19.7	14	21	23.3	**19A**	33	28.4	**19A**	50	36.0	**19A**	71	46.7	**19A**	92	48.2	**19A**	67	36.8	**19A**	54	27.4	**15A**	39	23.8
2	6B	52	15.5	**19A**	20	22.2	14	28	24.1	19F	19	13.7	19F	15	9.9	**15A**	18	9.4	**23B**	32	17.6	**15A**	40	20.3	**23B**	39	23.8
3	9V	45	13.4	19F	13	14.4	9V	15	12.9	6B	12	8.6	**23B**	13	8.6	19F	16	8.4	**15A**	27	14.8	**23B**	38	19.3	**19A**	28	17.1
4	**19A**	43	12.8	23F	12	13.3	23F	8	6.9	14	10	7.2	6B	10	6.6	**23B**	16	8.4	14	11	6.0	14	12	6.1	14	7	4.3
5	23F	40	11.9	9V	10	11.1	6B	8	6.9	23F	6	4.3	**15A**	9	5.9	9V	8	4.2	6A	7	3.8	19F	6	3.0	6B	6	3.7
6	19F	31	9.3	6B	5	5.6	19F	6	5.2	**15A**	5	3.6	14	6	3.9	14	5	2.6	19F	6	3.3	11A	5	2.5	6C	6	3.7
7	**15A**	7	2.1	6A	4	4.4	1	2	1.7	**23B**	5	3.6	9V	5	3.3	23F	5	2.6	6C	6	3.3	17F	4	2.0	24F	5	3.0
8	9A	7	2.1	24F	2	2.2	34	2	1.7	9V	5	3.6	23F	4	2.6	6B	4	2.1	6B	4	2.2	23F	4	2.0	35B	5	3.0
9	6A	5	1.5	15C	1	1.1	**15A**	2	1.7	18C	3	2.2	6A	4	2.6	17F	3	1.6	12F	3	1.6	24F	4	2.0	12F	4	2.4
10	4	4	1.2	35F	1	1.1	24F	2	1.7	6A	3	2.2	35B	3	2.0	22F	3	1.6	15B	3	1.6	6C	4	2.0	19F	4	2.4
1-10		300	89.6		89	98.9		106	91.4		118	84.9		140	92.1		170	89.0		166	91.2		171	86.8		143	87.2
>10	others	35	10.4	others	1	1.1	others	10	8.6	others	21	15.1	others	12	7.9	others	21	11.0	others	16	8.8	others	26	13.2	others	21	12.8
all		335	100.0		90	100.0		116	100.0		139	100.0		152	100.0		191	100.0		182	100.0		197	100.0		164	100.0
**Isolates**	**1992-2006**	**n=**	**%**	**2006-2007**	**n=**	**%**	**2007-2008**	**n=**	**%**	**2008-2009**	**n=**	**%**	**2009-2010**	**n=**	**%**	**2010-2011**	**n=**	**%**	**2011-2012**	**n=**	**%**	**2012-2013**	**n=**	**%**	**2013-2014**	**n=**	**%**
PenNS		335	5.1		90	5.9		116	5.8		139	6.2		152	6.8		191	7.5		182	8.1		197	8.2		164	7.4
all isolates		6552			1521			2008			2248			2228			2533			2241			2399			2227	

Serotypes 15A, 19A and 23B are depicted in bold for clarity.

### Multi locus sequence typing (MLST)

MLST was performed for 21 isolates with serotype 23B and 20 isolates with serotype 15A (due to budgetary restrictions MLST could not be performed on all isolates). Isolates were selected to cover as much of the surveillance period as possible, and covering both penicillin sensitive and PenNS isolates.

Serotype 23B isolates grouped in two clonal complexes: CC439, with ST439 as the main representative and CC338 (which is part of the very large CC156 complex), with ST1349 as most prevalent clone. Both CCs have been present over almost the whole surveillance period. All PenNS isolates occurred in CC338, whereas CC439 only contained isolates fully sensitive to penicillin (MIC = 0.015; Table 4).

**Table 4:** **Multi locus sequence types of serotype 15A (n = 20) and serotype 23B (n = 21) isolates, with their year of isolation and MIC towards penicillin**

**serotype**	**MLST**	**CC**	**aroE**	**gdh**	**gki**	**recP**	**Spi**	**xpt**	**ddl**	**n=**	**years**	**MIC PEN**
23B	439	439	1	8	9	2	6	4	6	7	1994,1998, 2001, 2007, 2008, 2009, 2010	0.015 (n = 7)
23B	**9867**	439	1	4	388	2	6	4	6	3	2012, 2013, 2014	0.015 (n = 3)
23B	**9872**	439	1	8	421	2	6	4	6	1	2003	0.015
23B	778	439	1	8	9	1	6	1	6	1	2006	0.015
23B	1349	338	18	13	8	6	3	6	8	6	1997, 2001, 2004, 2009, 2010, 2012	0.06; 0.12; 0.25 (n = 3); 0.5
23B	2372	338	18	13	8	6	3	6	46	3	2008, 2011 (n = 2)	0.015; 0.12; 0.25
15A	63	63	2	5	36	12	17	21	14	5	1998, 2001, 2006, 2008, 2009	0.06; 0.12 (n = 2); 0.25; 0.5
15A	2105	63	2	5	36	12	17	21	4	2	2010, 2013	1 (n = 2)
15A	2613	63	2	5	36	12	17	1	4	1	2013	2
15A	**9874**	63	2	5	422	12	17	21	14	1	2004	0.25
15A	3811	156	7	11	10	1	6	8	315	2	2013, 2014	0.015 (n = 2)
15A	**9868**	156	7	33	1	1	6	31	14	1	1999	0.015
15A	473	473	7	25	4	4	15	20	28	1	2002	0.015
15A	**9873**	473	7	25	4	4	15	20	623	1	2011	0.015
15A	410	193	8	10	2	16	7	26	1	1	2009	0.015
15A	199	199	8	13	14	4	17	4	14	1	2005	0.015
15A	1576	292	10	8	8	8	6	28	14	1	1998	0.12
15A	9308	group of three	15	5	15	1	398	1	18	1	2004	0.015
15A	**9871**	singleton	7	424	8	8	6	570	14	1	2012	0.015
15A	1577	singleton	10	13	67	16	6	1	8	1	1999	0.25

CC: clonal complex.

MLST in bold are new sequence types found for the first time in this study.

Serotype 15A isolates appeared to be more diverse. Six CCs, one group of three STs and two singletons were found among 20 isolates. Most prevalent was CC63, with ST63 as most prominent representative (n = 5). Most PenNS isolates were found among CC63 isolates (Table 4).

## Discussion

In Germany, 7-valent childhood pneumococcal conjugate vaccination has led to strong changes in the serotype distribution among IPD cases, with serotypes 7F and 19A as the main replacement serotypes. Introduction of higher-valent vaccines in 2009 has once again changed the serotype distribution, strongly reducing the role of the six additional serotypes in IPD. In the late vaccination (PCV13-) period, several non-vaccine serotypes have gained importance, among which serotypes 15A and 23B were the most strongly increasing.

Among children, a decrease in incidence of IPD after the introduction of PCV7 in Germany has been described [3,9]. In the higher-valent vaccine period, a further decrease is observed. This is in accordance with reports from other countries where PCV13 has been used [7,8].

Among adults, a strong herd protection effect on the serotype distribution was observed, with PCV7 serotypes disappearing in the early vaccination (PCV7) period. However, this decrease was off-set by an increase of PCV13-non-PCV7 serotype among this age group. In the late vaccination (PCV13) period, the prevalence of PCV13-non-PCV7 serotypes was strongly reduced, but now a full replacement by non-PCV13 serotypes has appeared. So far, a reduction in IPD among adults could not be shown, only a dramatic change in serotype distribution. A trivial reason for this could partially be the incompleteness of our surveillance system, with increased reporting off-setting the decrease in IPD due to vaccination. However, it seems unlikely that this would continue over the course of seven consecutive vaccination years and would stay in exact ‘pace’ with the reduction.

With Germany introducing PCV13 in December 2009, just before the beginning of the next winter season and almost half a year earlier than other countries (UK, USA (both April 2010)), effects of PCV13 on the serotype distribution and the settling of a new serotype equilibrium among IPD can be expected to appear in Germany first. The strong changes in prevalence of different members of serogroups 15 and 23 are a first indication of the establishment of a new equilibrium among the remaining, non-PCV13 serotypes.

The strong reduction of serotype 23F is no surprise, and was observed in all other countries where PCVs were introduced [6]. However, an increase in serotypes 23B and to a lesser extent 23A was unexpected. Several countries have reported increased prevalence of serotype 23B among carriage isolates [13-16], which could be a first indicator for an upcoming increase among IPD. Steens *et al.* reported an increase in serotypes 23B (and 15A) among IPD in Norway [7]. An interesting point is that there is apparently no cross-protection from 23F-antibodies towards either 23A or 23B. This once more underlines how different even the ‘related serotypes’ are from each other immunologically. Similarly, no cross-protection of 19F-antibodies raised by PCV7 towards serotype 19A has been observed [17,18]. Both clones of 23B that are increasing in Germany were already present in the pre-vaccination period, although they were very rare. This shows that we are witnessing the expansion of existing clones, rather than the import of new ones. The increase in 23B has been particularly strong in the late vaccination (PCV13) period, and seems to be stronger in adults than in children. The CC439 clone has been reported from several European countries, USA, Brazil, Tunisia and Australia, and therefore seems to be a worldwide spread clone, which is also always linked to penicillin susceptibility (http://pubmlst.org/spneumoniae/). ST1349 (CC338) was reported from European countries only (Greece, France, Turkey, Germany) and was regularly associated with penicillin non-susceptibility (http://pubmlst.org/spneumoniae/).

Increased prevalence of serotype 15A in the vaccination period has been reported from several countries, but also here, mostly from carriage and non-invasive disease isolates [7,19-22]. Serotype 15A has been very rare in Germany before the start of vaccination, and, like for serotype 23B, its increase is caused by an expansion of existing clones. The expansion has been particularly strong in the late vaccination (PCV13) period, and mostly among adults. ST63, the most prevalent 15A clone in this study, has been reported with serotypes 14 and 19A (Africa, Asia, Australia, Europe) and also with multi-drug resistance profiles, so the possibility of a serotype switch from these vaccine type serotypes is not unlikely (http://pubmlst.org/spneumoniae/).

The reported serotype 15A and 23B isolates show a high level of penicillin non-susceptibility. Even though reported MIC values are still, for the larger part, in the ‘intermediate resistant’ range (0.12-1 μg/ml), an evolution towards higher penicillin resistance levels is not unlikely. Even now, serotypes 15A and 23B are the most prevalent penicillin non-susceptible isolates in Germany. So far however, for invasive infections other than meningitis, the MIC-values are still in the susceptible range, indicating that these infections can still be treated successfully with beta-lactam antibiotics. In a case report from 2003, a coinfection of blood and CSF with serotypes 23F and 23B in a Brazilian child was described [23]. Both isolates were penicillin non-susceptible (MIC: 0.125), which is the same level as found among the 23B isolates in this study. Martin *et al.* report the emergence of penicillin non-susceptible serogroup 15 among isolates from children with acute otitis media [24].

A limitation of our study was that the referral of isolates to the reference center was incomplete, since reporting IPD is not mandatory in Germany. However, reported cases show no regional bias, and referral patterns have been constant for years. For children, using our capture-recapture incidence calculations, we determined that before the vaccination recommendation, 40-50% of all IPD cases had a sample sent to the GNRCS. This percentage increased to 50-60% after vaccination introduction [25].

## Conclusion

Our analysis shows that, after the introduction of higher-valent pneumococcal conjugate vaccination in Germany, the serotype distribution among IPD has changed dramatically once more. Clones of serotypes 15A and 23B, which were rarely reported in the pre-vaccination period, have started to increase. The increase was mainly in the late vaccination (PCV13) period and therefore seems to be an effect of higher-valent (PCV13) vaccination. A most worrisome fact is that almost half of the reported isolates of these two serotypes are penicillin non-susceptible, and, for serotype 15A, even multi-drug resistant. This is a situation reminiscent of the increase of serotype 19A reported in many countries after the introduction of PCV7 [26-28], and underlines the need for further careful monitoring of the impact of conjugate vaccines on the pneumococcal population.

## References

[1] Pneumococcal conjugate vaccine for childhood immunization--WHO position paper. Wkly Epidemiol Rec 2007, 82(12):93-104. 17380597

[2] Whitney CG, Farley MM, Hadler J, Harrison LH, Bennett NM, Lynfield R (2003). Decline in invasive pneumococcal disease after the introduction of protein-polysaccharide conjugate vaccine. N Engl J Med.

[3] van der Linden M, Weiss S, Falkenhorst G, Siedler A, Imohl M, von Kries R (2012). Four years of universal pneumococcal conjugate infant vaccination in Germany: impact on incidence of invasive pneumococcal disease and serotype distribution in children. Vaccine.

[4] Miller E, Andrews NJ, Waight PA, Slack MP, George RC (2011). Herd immunity and serotype replacement 4 years after seven-valent pneumococcal conjugate vaccination in England and Wales: an observational cohort study. Lancet Infect Dis.

[5] Lepoutre A, Varon E, Georges S, Dorleans F, Janoir C, Gutmann L (2015). the Microbiologists of the E, the ORPN: Impact of the pneumococcal conjugate vaccines on invasive pneumococcal disease in France, 2001-2012. Vaccine.

[6] Feikin DR, Kagucia EW, Loo JD, Link-Gelles R, Puhan MA, Cherian T (2013). Serotype-Specific Changes in Invasive Pneumococcal Disease after Pneumococcal Conjugate Vaccine Introduction: A Pooled Analysis of Multiple Surveillance Sites. PLoS Med.

[7] Steens A, Bergsaker MA, Aaberge IS, Ronning K, Vestrheim DF (2013). Prompt effect of replacing the 7-valent pneumococcal conjugate vaccine with the 13-valent vaccine on the epidemiology of invasive pneumococcal disease in Norway. Vaccine.

[8] Miller E, Andrews NJ, Waight PA, Slack MP, George RC (2011). Effectiveness of the new serotypes in the 13-valent pneumococcal conjugate vaccine. Vaccine.

[9] Rückinger S, van der Linden M, Reinert RR, von Kries R, Burckhardt F, Siedler A (2009). Reduction in the incidence of invasive pneumococcal disease after general vaccination with 7-valent pneumococcal conjugate vaccine in Germany. Vaccine.

[10] Institute CLS (2014). Performance Standards for Antimicrobial Susceptibility Testing; Twenty-Fourth Informational Supplement.

[11] Enright MC, Spratt BG (1998). A multilocus sequence typing scheme for *Streptococcus pneumoniae* identification of clones associated with serious invasive disease. Microbiology.

[12] Francisco AP, Vaz C, Monteiro PT, Melo-Cristino J, Ramirez M, Carrico JA (2012). PHYLOViZ: phylogenetic inference and data visualization for sequence based typing methods. BMC Bioinformatics.

[13] Grivea IN, Priftis KN, Giotas A, Kotzia D, Tsantouli AG, Douros K (2014). Dynamics of pneumococcal carriage among day-care center attendees during the transition from the 7-valent to the higher-valent pneumococcal conjugate vaccines in Greece. Vaccine.

[14] Richter SS, Diekema DJ, Heilmann KP, Dohrn CL, Riahi F, Doern GV (2014). Changes in pneumococcal serotypes and antimicrobial resistance after introduction of the 13-valent conjugate vaccine in the United States. Antimicrob Agents Chemother.

[15] Almeida ST, Nunes S, Santos Paulo AC, Valadares I, Martins S, Breia F (2014). Low prevalence of pneumococcal carriage and high serotype and genotype diversity among adults over 60 years of age living in Portugal. PLoS One.

[16] Ricketson LJ, Wood ML, Vanderkooi OG, MacDonald JC, Martin IE, Demczuk WH (2014). Trends in asymptomatic nasopharyngeal colonization with streptococcus pneumoniae after introduction of the 13-valent pneumococcal conjugate vaccine in Calgary, Canada. Pediatr Infect Dis J.

[17] Grant LR, O'Brien SE, Burbidge P, Haston M, Zancolli M, Cowell L (2013). Comparative immunogenicity of 7 and 13-valent pneumococcal conjugate vaccines and the development of functional antibodies to cross-reactive serotypes. PLoS One.

[18] Hausdorff WP, Hoet B, Schuerman L (2010). Do pneumococcal conjugate vaccines provide any cross-protection against serotype 19A?. BMC Pediatr.

[19] Lee GM, Kleinman K, Pelton SI, Hanage W, Huang SS, Lakoma M (2014). Impact of 13-Valent Pneumococcal Conjugate Vaccination on Carriage in Young Children in Massachusetts. J Pediatr Infect Dis Soc.

[20] Parra EL, De La Hoz F, Diaz PL, Sanabria O, Realpe ME, Moreno J (2013). Changes in Streptococcus pneumoniae serotype distribution in invasive disease and nasopharyngeal carriage after the heptavalent pneumococcal conjugate vaccine introduction in Bogota, Colombia. Vaccine.

[21] McElligott M, Vickers I, Cafferkey M, Cunney R, Humphreys H (2014). Non-invasive pneumococcal serotypes and antimicrobial susceptibilities in a paediatric hospital in the era of conjugate vaccines. Vaccine.

[22] Liyanapathirana V, Nelson EA, Ang I, Subramanian R, Ma H, Ip M (2015). Emergence of serogroup 15 Streptococcus pneumoniae of diverse genetic backgrounds following the introduction of pneumococcal conjugate vaccines in Hong Kong. Diagn Microbiol Infect Dis.

[23] de Andrade AL, Pimenta FC, Brandileone MC, Laval CA, Guerra ML, de Andrade JG (2003). Genetic relationship between Streptococcus pneumoniae isolates from nasopharyngeal and cerebrospinal fluid of two infants with Pneumococcal Meningitis. J Clin Microbiol.

[24] Martin JM, Hoberman A, Paradise JL, Barbadora KA, Shaikh N, Bhatnagar S (2014). Emergence of Streptococcus pneumoniae Serogroups 15 and 35 in Nasopharyngeal Cultures From Young Children With Acute Otitis Media. Pediatr Infect Dis J.

[25] van der Linden M, von Kries R, Imöhl M, Rückinger S, Reinert R (2010). Increased disease awareness after onset of a national immunization program with 7-valent pneumococcal conjugate vaccine. 7th International Symposium on Pneumococci & Pneumococcal Diseases.

[26] Kaplan SL, Barson WJ, Lin PL, Stovall SH, Bradley JS, Tan TQ (2010). Serotype 19A Is the most common serotype causing invasive pneumococcal infections in children. Pediatrics.

[27] Fenoll A, Granizo JJ, Aguilar L, Gimenez MJ, Aragoneses-Fenoll L, Hanquet G (2009). Temporal trends of invasive Streptococcus pneumoniae serotypes and antimicrobial resistance patterns in Spain from 1979 to 2007. J Clin Microbiol.

[28] Mahjoub-Messai F, Doit C, Koeck JL, Billard T, Evrard B, Bidet P (2009). Population snapshot of Streptococcus pneumoniae serotype 19A isolates before and after introduction of seven-valent pneumococcal Vaccination for French children. J Clin Microbiol.

